# Influence of Heat Treatment on Microstructure, Mechanical Properties, and Damping Behavior of 2024 Aluminum Matrix Composites Reinforced by Carbon Nanoparticles

**DOI:** 10.3390/nano14161342

**Published:** 2024-08-14

**Authors:** Wilson Rativa-Parada, Sabrina Nilufar

**Affiliations:** School of Mechanical, Aerospace, and Materials Engineering, Southern Illinois University, Carbondale, IL 62901, USA

**Keywords:** 2024 aluminum composites, heat treatment, nanocarbon allotropes, damping properties

## Abstract

Nanocarbon 2024 aluminum composites with 0.5 vol. % and 1 vol. % of graphene nanoplatelets and 1 vol. % and 2 vol. % of activated nanocarbon were manufactured through induction casting. The effect of the reinforcements and heat treatment on the performance of the composites was examined. Analysis of the microstructure of the composites before heat treatment suggested the homogeneous dispersion of reinforcements and the absence of secondary carbide or oxide phases. The presence of carbon nanoparticles had a significant impact on the microstructural characteristics of the matrix. This behavior was further enhanced after the heat treatment. The mechanical and damping properties were evaluated with the uniaxial compression test, micro Vickers hardness test, and dynamic mechanical analysis. The yield strength and ultimate strength were improved up to 28% (1 vol. % of graphene nanoplatelets) and 45% (0.5 vol. % of graphene nanoplatelets), respectively, compared to the as-cast 2024 aluminum. Similarly, compared to the heat-treated 2024 aluminum, the composites increased up to 56% (0.5 vol. % of graphene nanoplatelets) and 57% (0.5 vol. % of graphene nanoplatelets) in yield strength and ultimate strength, respectively. Likewise, the hardness of the samples was up to 33% (1 vol. % of graphene nanoplatelets) higher than that of the as-cast 2024 aluminum, and up to 31% (2 vol. % of activated nanocarbon) with respect to the heat-treated 2024 aluminum. The damping properties of the nanocarbon–aluminum composites were determined at variable temperatures and strain amplitudes. The results indicate that damping properties improved for the composites without heat treatment. As a result, it is demonstrated that using small volume fractions of nanocarbon allotropes enhanced the mechanical properties for both with- and without-heat treatment with a limited loss of plastic deformation before failure for the 2024 aluminum matrix.

## 1. Introduction

Within the whole family of aluminum alloys, 2024 aluminum is well known because of its high strength and fatigue resistance. Aluminum matrix composites have emerged as an alternative in the search for stronger and lighter 2024 aluminum for the aerospace and automobile industries. Aluminum matrix composites have excelled because of their outstanding mechanical, structural, and corrosion properties [1,2,3,4,5,6,7]. Studies on composites based on 2024 aluminum matrix composites have increased in number in recent decades, with promising results [8,9,10,11,12,13]. For instance, Jiang et al. [14] increased the yield and ultimate strength of 2024 aluminum matrix composites after using 5 vol. % of Al_2_O_3_ as reinforcement. Their results were attributed to the uniform dispersion of the reinforcement nanoparticles and the high dislocation density. Also, Yuan et al. [15] utilized high-entropy alloy particles of CoCrFeMnNi as reinforcement for 2024 aluminum. The study revealed that the formation of a diffusion layer increased the hardness of the composite because of the new phase formation at the interface of the high-entropy alloy particle and aluminum alloy matrix. Xie et al. obtained 2024 aluminum matrix composites with 0.8 vol. % of nanodiamond particles, wherein the reinforcement was homogeneously dispersed by applying a surface modification procedure, also resulting in an enhanced ultimate tensile strength of up to more than 40% compared to conventional 2024 aluminum [16].

Similarly, heat treatment has demonstrated a high influence on the performance of aluminum composites. Zhang et al. [17] used 1.5 wt. % of Al_2_O_3_ to reinforce aluminum alloy matrix composites with T6 heat treatment. In that work, the materials presented an enhanced hardness compared to untreated composites, because of phase transformations. Likewise, Mistry et al. [18] published a study on the behavior of heat-treated aluminum matrix composites after being combined with Si_3_N_4_ particles by a stir-casting method. These materials presented improved hardness, flexural strength, and tensile strength at 8 wt. %. However, higher volume fractions had a negative impact on the mechanical properties due to a clustering tendency of Si_3_N_4_ particles and a weak interface. Hanizam et al. [19] utilized a liquid method to obtain carbon nanotube aluminum alloy matrix composites subjected to T6 heat treatment. Enhanced ultimate tensile strength and hardness were attributed to load transfer strengthening and improved wettability. However, this was limited to 1 wt. % of carbon nanotube reinforcement. Lakshmikanthan et al. [20] assessed the tribological properties of aluminum matrix composites reinforced with silicon carbide. After T6 heat treatment, the materials presented the formation of secondary phases of Mg_2_Si, which contributed to the increase in the wear and mechanical properties of the composites. 

Aluminum composites also have the potential to boost the damping properties of pristine aluminum alloys. In this regard, Rojas et al. [21] studied the viscoelastic response of SiC aluminum matrix composites. In that report, they obtained materials with higher damping friction produced by hindering the sliding of dislocations of the matrix, typically occurring at the grain boundary, due to the localization of reinforcements in these specific zones. Hu et al. [22] also investigated the damping behavior of aluminum matrix composites with 20 vol. % of TiNi and variable volume fractions of silicon carbide, finding that phase transformations of TiNi at high temperatures can increase the damping properties of these materials. However, the mechanisms behind heat-treated 2024 aluminum matrix composites with simultaneous improved damping and mechanical properties remained little explored, limiting their industrial implementation. 

Based on this, this work aimed to establish the manufacturing conditions required to obtain 2024 aluminum matrix composites with superior mechanical properties and enhanced damping properties after the introduction of small volume fractions of activated nanocarbon and graphene nanoplatelets by studying their structural evolution, mechanical performance, and damping performance before and after heat treatment.

## 2. Materials and Methods

### 2.1. Composites Preparation

Activated nanocarbon (<100 nm, 95%) and graphene nanoplatelets (99.5%) were purchased from US Research Nanomaterials, Inc. (Houston, TX, USA). 2024 aluminum with the following composition was utilized: Al = 90.75–94.7%, Cr = 0–0.1%, Cu = 3.8–4.9%, Fe = 0–0.5%, Mg = 1.2–1.8%, Mn = 0.3–0.9%, Si = 0–0.5%, Ti = 0–0.15%, Zn = 0–0.25%. The nanocarbon 2024 aluminum composites were prepared via the induction casting method. Corresponding amounts of 2024 aluminum were introduced into a SiC crucible, which was placed inside an induction furnace (RDO Model DuraPower, 10 kW, 20–80 kHz, 220 V, 3-Phase) (Washington, NJ, USA) under an argon atmosphere. The system was heated up to 750 °C, and once the alloy was completely melted, corresponding amounts of activated nanocarbon and graphene nanoplatelets were introduced into the crucible. The system was maintained at 750 °C for 30 min. After that, the composite was poured into a cast iron mold to solidify. Selected samples were subjected to T4 heat treatment. First, the samples were heated up to 495 °C for 70 min, followed by an immediate quenching in water at 30 °C. After this, the composites were naturally aged. The denomination of the samples after manufacturing is shown in Table 1.

### 2.2. Structural Characterization

All the samples were ground with abrasive papers (80 to 1200) and polished with an alumina suspension. The microstructures, distribution of reinforcements, and fracture surfaces were observed with scanning electron microscopy (Quanta FEG 450) (Waltham, MA, USA) coupled with energy-dispersive X-ray spectroscopy (EDS) (Concord, MA, USA). The XRD analysis was performed with Cu radiation (λ = 1.5405 Å) with a voltage of 30 KV and between 10 and 90 degrees, using a Rigaku D/Max-B diffractometer (The Woodlands, TX, USA). Density was calculated employing the Archimedes principle in distilled water. 

### 2.3. Mechanical Characterization

Uniaxial compressive tests were performed in an MTS Insight 30 kN standard length machine (Eden Prairie, MN, USA) following the ASTM E9 [23] for samples with a 2:1 height-to-width ratio (3 tests per sample). The average yield strength and the ultimate strength of the materials were obtained from the stress–strain diagrams. A total of 10 indentations per sample were used to obtain the hardness of the materials with an HMV-G31 DT micro Vickers tester from Shimadzu Scientific Instruments, Inc. (Pittsburgh, PA, USA) equipped with a diamond indenter. A load of 1.961 N for 15 s was employed each time.

### 2.4. Dynamic Mechanical Analysis

Damping properties were assessed with dynamic mechanical analysis (Discover DMA 850) (New Castle, DE, USA), using a single cantilever configuration, from room temperature to 400 °C at 10 Hz, and a heating rate of 5 °C min^−1^ in a Nitrogen atmosphere. The samples were also tested against a strain amplitude between 1 × 10^−3^ to 0.1%.

## 3. Results and Discussion

### 3.1. Structural Characterization

**Figure 1:**
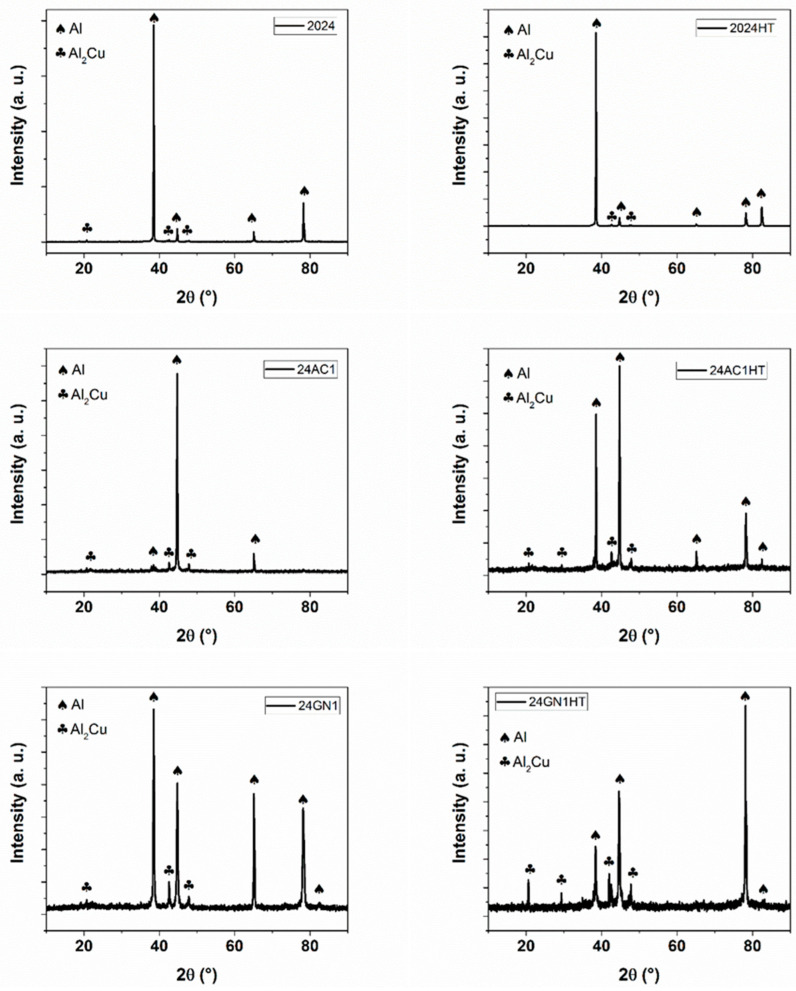
XRD patterns of the nanocarbon 2024 aluminum composites with and without heat treatment.

Figure 1 shows the results of the XRD characterization of the nanocarbon 2024 aluminum matrix composites. The typical phases of aluminum and CuAl_2_ of the metal matrix can be seen, before and after heat treatment, and without any reinforcement. Introducing either activated nanocarbon or graphene nanoplatelets did not alter the presence and formation of these two phases. Therefore, the peaks located at 38.4°, 44.7°, 65.3°, and 78.5° were attributed to the planes (1 1 1), (2 0 0), (2 2 0), and (3 1 1) of aluminum, respectively [24]. The peaks at 20.7°, 29.3°, 44.3°, and 47.3° were ascribed to the phase CuAl_2_ with the planes (1 1 0), (2 0 0), (1 1 2), and (1 3 0), respectively [14]. Similarly, no peaks were associated with carbon or carbide structures. The intensity of the peaks varies due to the occurrence of preferential orientation. The crystallite sizes of the composites were determined with Equation (1) to distinguish between the influence of nanoparticles and heat treatment on the microstructural behavior of the materials.

(1) $$D=\frac{0.9\lambda }{\beta \;cos\;\theta }$$

where *λ* = 1.5405 Å, *D* is crystallite size, *θ* is the diffraction angle, and *β* is Full Width at Half Maximum (FWHM).

According to these results, the crystallite size of the materials decreased for both carbon nanoparticles as a function of the volume fraction, and the heat treatment improved this tendency. So, the crystallite sizes of the samples 2024, 2024-HT, 24AC1, and 24GN1 were 37.1, 32.2, 24.1, and 24.7 nm, respectively. This indicates the effect that these nanocarbon reinforcements have on the refinement of the composite structures compared to the original matrix and the heat treatment without any reinforcement, by following the grain refinement strengthening mechanism generated by the increase in the dislocation density [25]. Similarly, the crystallite sizes of the heat-treated samples 24AC1-HT and 24GN1-HT were 23.3 and 22.5 nm, respectively.

**Figure 2:**
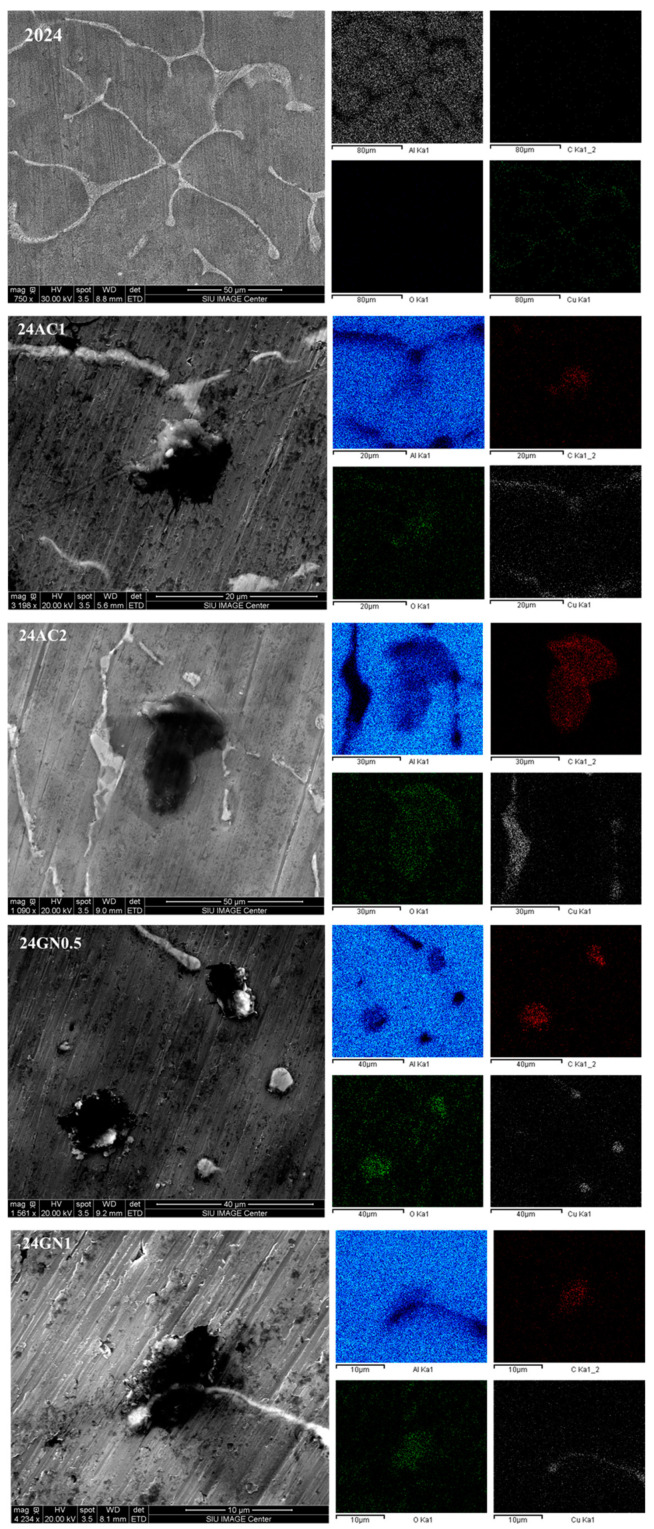
SEM of the samples 2024, 24AC1, 24AC2, 24GN0.5, and 24GN1 showing the elemental composition and the presence of the nanocarbon reinforcement.

**Figure 3:**
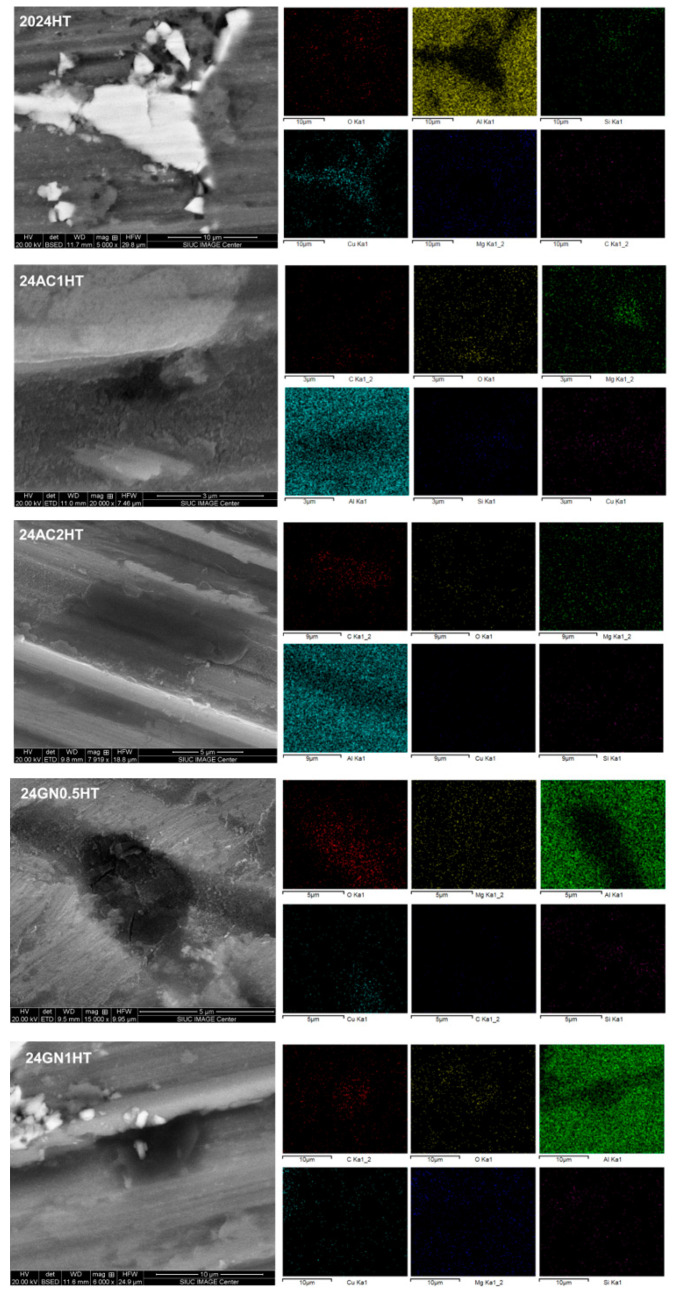
SEM of the samples 2024-HT, 24AC1-HT, 24AC2-HT, 24GN0.5-HT, and 24GN1-HT showing the elemental composition and the presence of the nanocarbon reinforcement.

Figure 2 shows the SEM images of the as-cast composites 2024, 24GN0.5, 24GN1, 24AC1, and 24AC2. The carbon nanoparticle fillers presented a homogenous distribution in the composite microstructure. Energy-dispersive X-ray spectroscopy (EDS) images of the as-cast composites revealed that the reinforcement/matrix interface comprises only carbon nanoparticles of graphene nanoplatelets and activated nanocarbon, and 2024 aluminum, without the formation of intermediate phases such as Al_4_C_3_, following the XRD results shown above. With EDS, some bright zones rich in copper and magnesium were also detected all over the surface, typical for the composition of this aluminum alloy [26,27]. Similarly, the images of the samples after heat treatment are shown in Figure 3, in which the presence of precipitates was also evident, but without altering the dispersion tendency shown by the nanocarbon reinforcements in the as-cast composites. It was also confirmed that the carbon allotropes did not only react with aluminum to form carbide phases but also did not react with the alloy elements to form phases that could alter the formation of the typically expected precipitates for this 2024 aluminum. This differs from some other reports in which the reinforcement, even at a low volume fraction, (i.e., 2 vol. % of TiC) reacted with the main elements of the alloy (i.e., Si) to form secondary phases (Al_3_TiSi_x_C_y_), which had an adverse effect for the thermal stability of the reinforcement and the optimal strength and plastic deformation of the composite [28].

### 3.2. Mechanical Characterization

**Figure 4:**
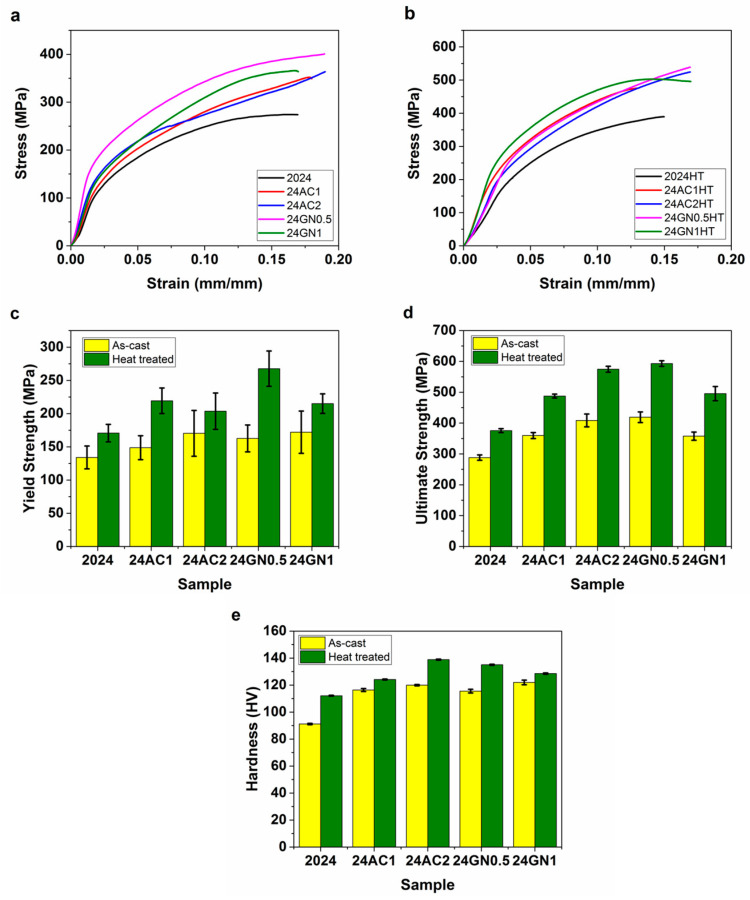
Representative stress–strain diagrams of (**a**) the nanocarbon 2024 aluminum composites, (**b**) the nanocarbon 2024-HT aluminum composites, and the average (**c**) yield strength, (**d**) ultimate strength, and (**e**) hardness of the nanocarbon 2024 aluminum composites and the nanocarbon 2024-HT aluminum composites.

Representative strain–stress curves of the composites before and after heat treatment are displayed in Figure 4a,b. Figure 4c,d shows the compressive properties of the as-cast and heat-treated materials reinforced with activated nanocarbon and graphene nanoplatelets. The samples presented higher values for yield strength and ultimate strength. The yield strength of the as-cast composites was 27% and 28% higher than that of unreinforced 2024 aluminum for the composites 24AC2 and 24GN1, respectively. Heat treatment also increased the yield strength by 26% compared to the pure 2024 aluminum without heat treatment. Similarly, the heat-treated composites 24AC1-HT and 24GN0.5-HT obtained a yield strength of 28% and 56%, respectively, higher than that of the heat-treated 2024 aluminum. Likewise, the ultimate strength of the as-cast materials increased by 45% and 41% for the samples 24GN0.5 and 24AC2, respectively, compared to the as-cast 2024 aluminum. Moreover, compared to the heat-treated 2024 aluminum, the composites 24AC2-HT and 24GN0.5-HT increased by 52% and 57%, respectively. These yield and ultimate strength values decreased with the amount of graphene nanoplatelets due to a higher tendency to agglomerate and a larger porosity when inside the composites [5]. In addition, these values increased after heat treatment due to the formation and presence of precipitates, which limited the movement of dislocations, increased the dislocation density, and increased the strain hardening [29].

The micro Vickers hardness results for the composites as a function of the nanocarbon fraction and before and after the heat treatment are shown in Figure 4e. First, the hardness of the as-cast composites increased with the addition of both activated nanocarbon and graphene nanoplatelets. The hardness increased by 27% and 31% for the samples 24AC1 and 24AC2, respectively, whereas it was boosted by 26% and 33% for the samples 24GN0.5 and 24GN1, respectively, compared to the un-reinforced 2024 aluminum. After heat treatment, the samples continued to increase in hardness. The composites presented an enhancement of 17% and 31% for the materials 24AC1-HT and 24AC2-HT, as well as 28% and 21% for the materials 24GN0.5-HT and 24GN1-HT compared to the heat-treated 2024 aluminum. As mentioned above, heat treatment led to the formation of small precipitates, mainly of CuAl_2_, which increased the internal stresses and the dislocation density around them and limited the plastic deformation [30]. 

### 3.3. Dynamic Mechanical Analysis Results

**Figure 5:**
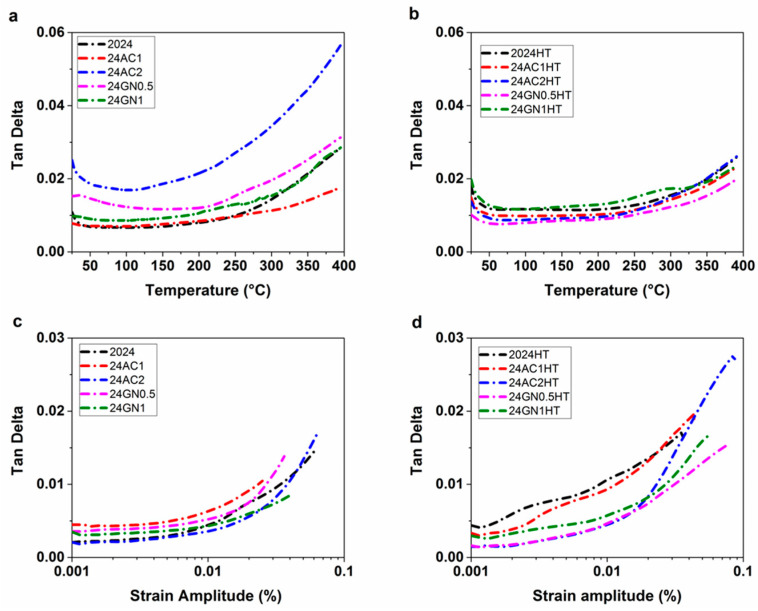
The DMA of (**a**) nanocarbon 2024 aluminum composites against temperature, (**b**) nanocarbon 2024-HT aluminum composites against strain amplitude, (**c**) nanocarbon 2024 aluminum composites against strain amplitude, and (**d**) nanocarbon 2024-HT aluminum composites against strain amplitude.

The DMA for the composites before and after heat treatment are shown in Figure 5. The tan delta vs. temperature curves for the as-cast materials show that the damping capacity of the composites increased at low temperatures after introducing the carbon nanoparticle fillers, as shown in Figure 5a. This behavior remained almost unchanged in the temperature range of 25 °C to 200 °C. This tendency changed at higher temperatures, in which the damping capacities increased at a higher rate. Damping occurs mainly by three mechanisms: the intrinsic damping properties of the reinforcement, matrix/reinforcement interface, and dislocations [31,32,33]. This improvement in damping properties has been associated with an effective transfer of the inherent high damping capacities of the nanocarbon reinforcements and the movement and friction of the dislocations at low temperatures, complemented by an enhanced energy dissipation capacity at the metal/nanocarbon interfaces and energy-activated grain boundary damping at high temperatures [34]. As shown in Figure 5b, the damping capacity of the materials after the heat treatment was reduced mainly because of the reduction in dislocation density after the solution and aging treatments, showing that the post-processing heat treatment reduces the damping properties of the nanocarbon-reinforced 2024 aluminum composites. Likewise, Figure 5c,d shows that the internal friction of the composites increased with the increase in strain amplitude before and after heat treatment. As the materials follow the damping dislocation mechanism, a higher amount of dislocation slipping was carried out in the composites with the increasing strain amplitude [35]. Therefore, the higher the strain amplitude, the higher the capacity of the composites to dissipate strain energy. As happened with the samples tested as a function of the temperature, the damping behavior of the composites relied on the dislocation density and the effect of the heat treatment on the matrix.

### 3.4. Density Results

**Figure 6:**
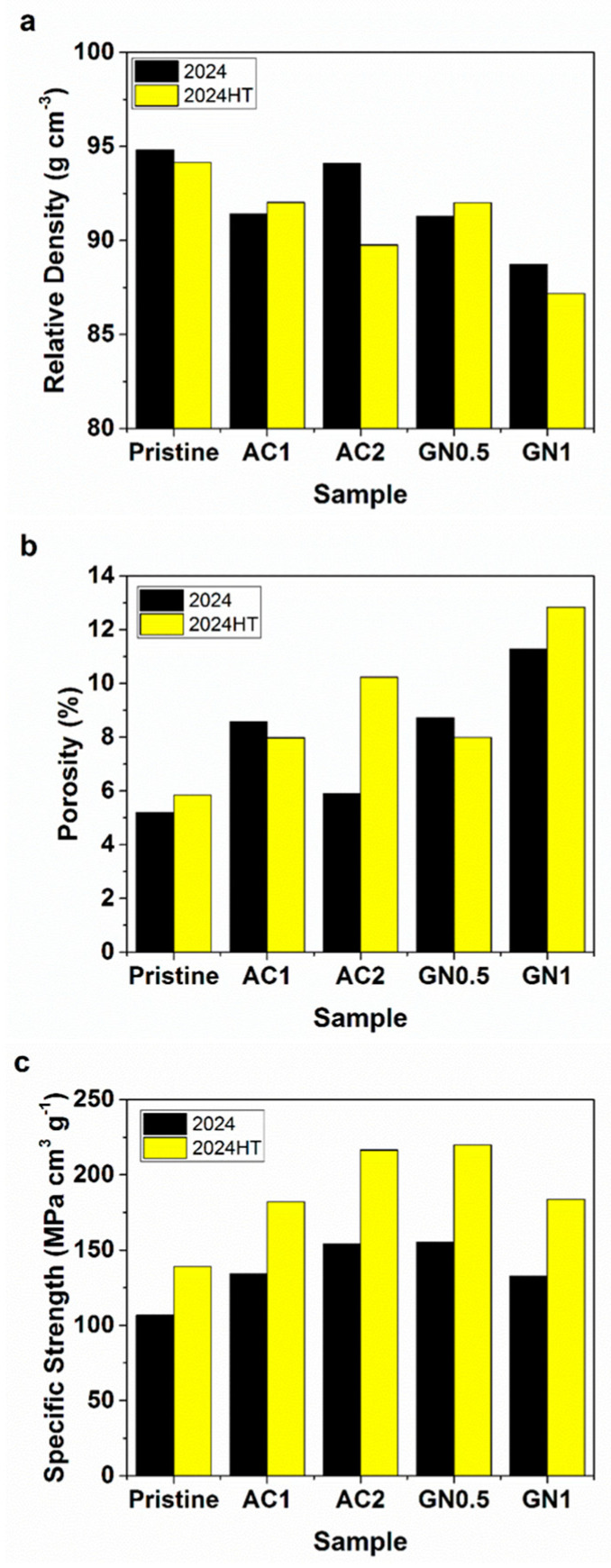
(**a**) Relative density, (**b**) porosity, and (**c**) specific strength of nanocarbon 2024 aluminum composites and nanocarbon 2024-HT aluminum composites.

Figure 6 displays the relative density, porosity, and specific strength of the composites with and without heat treatment. It shows that the density of the samples decreased with the volume fraction of the reinforcements due to the lower density of activated nanocarbon and graphene nanoplatelets. The relative density of the materials, in Figure 6a, was closer to the theoretical values for these compositions, calculated with Equation (2). This differs from other reinforcements, such as high-entropy alloys [36], and TiO_2_ [37], in which the density of the composites increased with the volume fraction of the reinforcement compared to the base matrix. Likewise, the porosity of the composites was determined using Equation (3), which shows that it increased with the incorporation of carbon nanoparticles as a consequence of the manufacturing method, and it influenced the relative density of the composites as the volume fraction of reinforcements was changing.

(2) $$\rho_{c}=V_{m}\rho_{m}+V_{r}\rho_{r}$$

where *ρ_m_* and *ρ_r_* are the density of the matrix and reinforcement, respectively, and *V_r_* and *V_m_* are the volume fraction of the reinforcement and matrix, respectively.

(3) $$Porosity\;\left(\%\right)=\frac{\rho_{t}-\rho_{e}}{\rho_{t}}\times 100\%$$

where *ρ_t_* is the theoretical density of the composite and *ρ_e_* is the experimental density of the composites.

The specific strength of these composites before and after heat treatment was also calculated, as shown in Figure 6c. It increased with the incorporation of activated nanocarbon and graphene nanoplatelets, and this resulted in higher values for the heat-treated samples. Therefore, the specific strength increased with the volume fraction of activated nanocarbon but decreased with the volume fraction of graphene nanoplatelets, following the tendencies observed for the yield strength, ultimate strength, and hardness of the samples.

### 3.5. Fracture Surface Characterization

**Figure 7:**
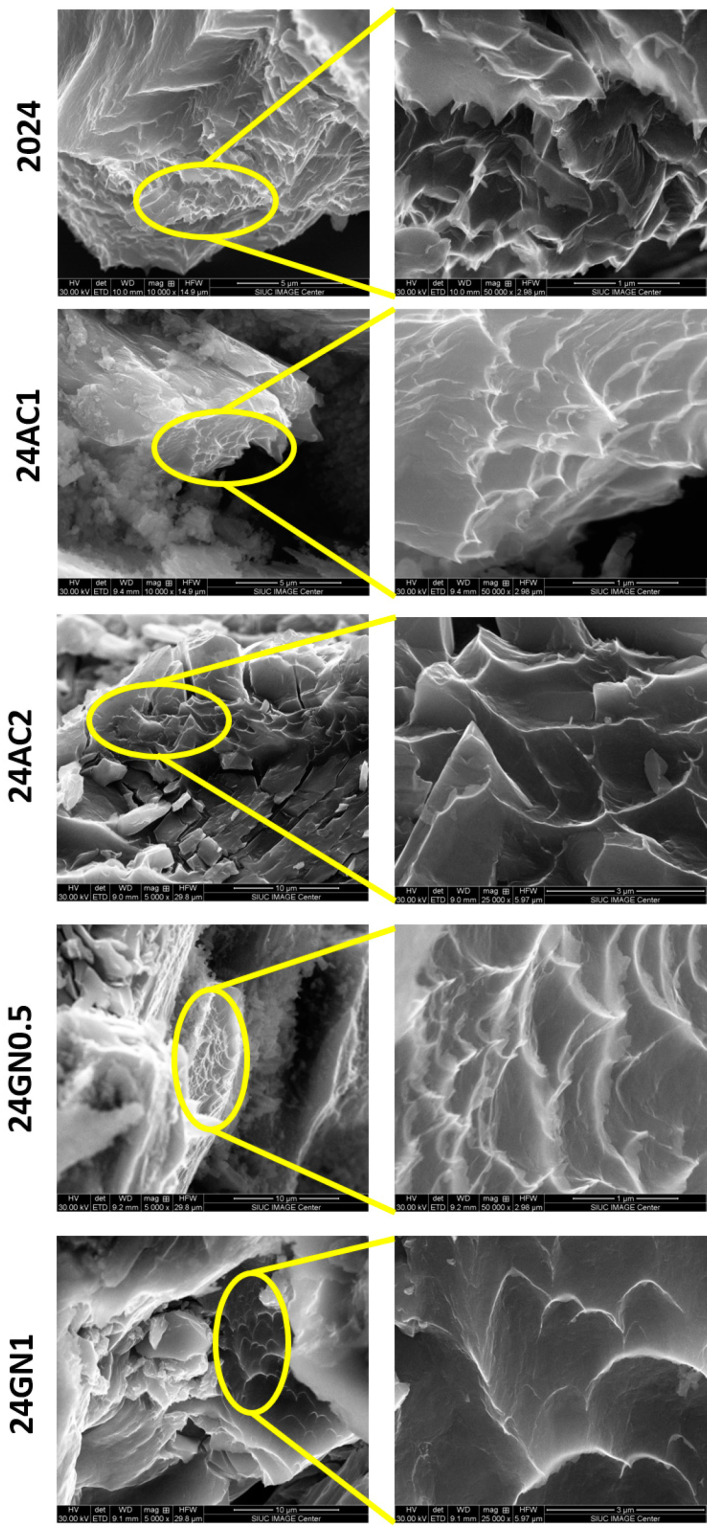
SEM of the samples 2024, 24AC1, 24AC2, 24GN0.5, and 24GN after fracture.

**Figure 8:**
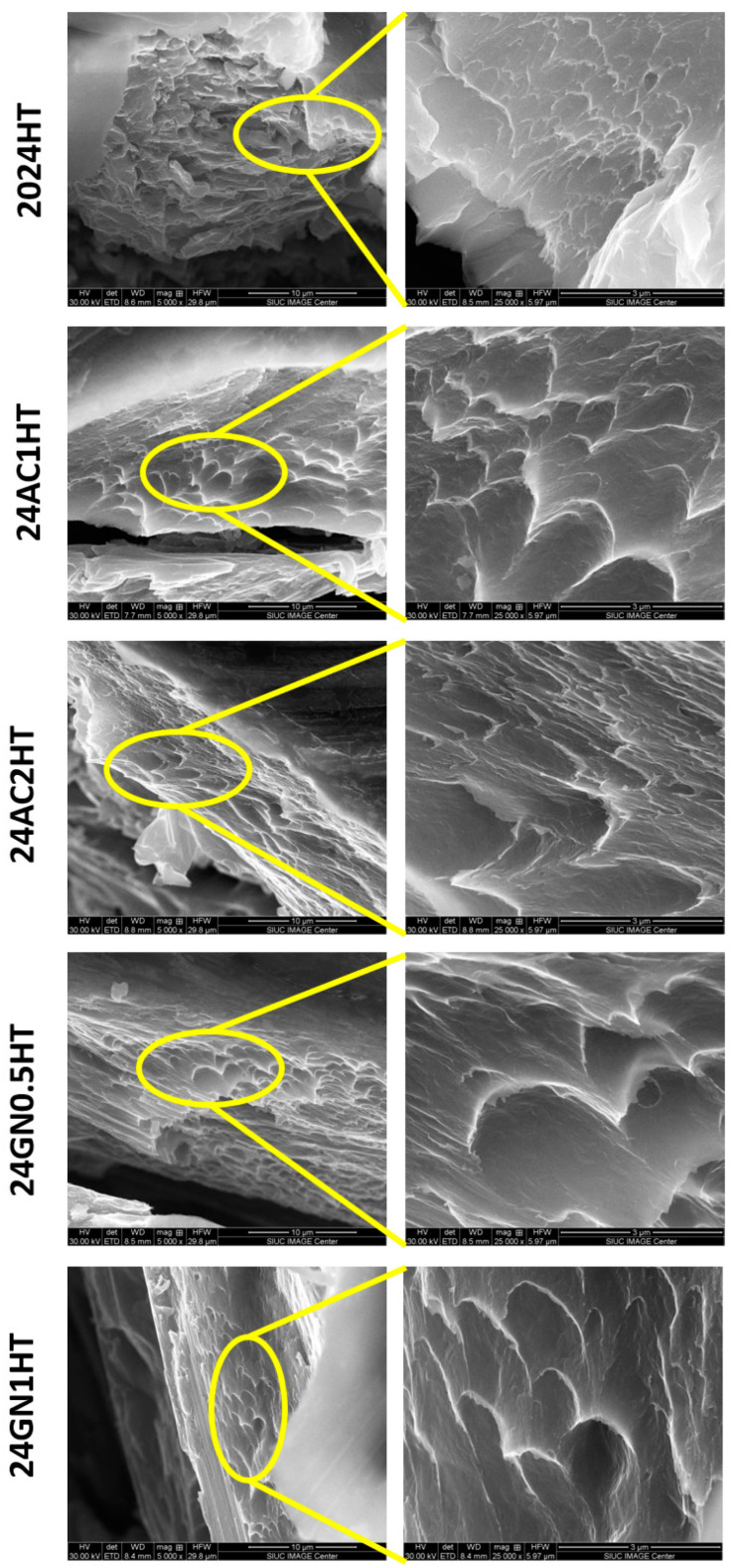
SEM of the samples 2024-HT, 24AC1-HT, 24AC2-HT, 24GN0.5-HT, and 24GN-HT after fracture.

The fractured surfaces of the representative compression test samples with 0.5 vol. % and 1 vol. % of graphene nanoplatelets and 1 vol. % and 2 vol. % of activated nanocarbon before and after the heat treatment can be observed in Figure 7 and Figure 8, respectively. The SEM micrographs of all the samples presented a large number of dimples and ridges of different sizes as well as little presence of flat planes inside the composites after the compressive loading process, which is the result of the nucleation, growth, and propagation of cracks near the micropores and interfaces inside the composites. In addition, signs of de-bonding and pulling-out of carbonaceous particles as a consequence of the mechanical bonding between matrix and reinforcements can be seen. This behavior agreed with the results presented in Figure 4 about the limited loss of elongation in the composites, typical of the ductile failure mechanism, due to the small size of the reinforcement and their good distribution inside the matrix. This differs from the results for solid manufacturing processes, in which the presence of dimples, ridges, and total elongation is reduced with the increasing introduction of reinforcements [38].

## 4. Conclusions

2024 aluminum composites reinforced with small volume fractions of activated nanocarbon and graphene nanoplatelets were successfully obtained by the induction casting process. The effect of reinforcement and heat treatment on the microstructure, mechanical properties, and damping behavior was assessed. The as-cast materials presented a good distribution of the carbon nanoparticles without evidencing the reaction between matrix and reinforcement to form interfacial phases. The characterization with XRD confirmed the effect of small volume fractions of the reinforcements on the refinement of the matrix microstructure. The yield strength increased by a maximum of 28% for the sample 24GN1, and the ultimate strength increased by a maximum of 45% for the sample 24GN0.5 compared to the as-cast 2024 aluminum. The enhancement of the heat-treated samples was superior for the sample 24GN0.5-HT, which presented a yield strength and ultimate strength of 56% and 57%, respectively, higher than those of the heat-treated 2024 aluminum. Likewise, the maximum values for hardness for the samples without heat treatment were obtained for the composition 24GN1, which was 33% higher than the as-cast 2024 aluminum. In turn, the hardness of the sample 24AC2-HT presented the biggest increment of 31% regarding the heat-treated 2024 aluminum. Nanocarbon allotropes and heat treatment also influenced the damping behavior. The damping properties of the as-cast composites were enhanced by the intrinsic damping properties of carbon nanoparticles, damping of dislocations, and the internal friction of both grain boundaries and interfaces of the composites as a function of temperature and strain amplitude. The heat treatment reduced dislocation density, which decreased the contribution of carbon nanoparticles and precipitates on the damping behavior. As a result, it has been demonstrated that carbon allotrope reinforcements, matrix structure, and manufacturing conditions can be tailored to obtain 2024 aluminum composites with enhanced mechanical and damping properties for industrial use.

## Figures and Tables

**Table 1:** Nominal composition of the samples.

Denomination	Composition
2024	2024 aluminum without any reinforcement
24Ac1	2024 aluminum reinforced with 1 vol. % of activated nanocarbon
24Ac2	2024 aluminum reinforced with 2 vol. % of activated nanocarbon
24Gn0.5	2024 aluminum reinforced with 0.5 vol. % of graphene nanoplatelets
24Gn1	2024 aluminum reinforced with 1 vol. % of graphene nanoplatelets
2024-HT	2024 aluminum without any reinforcement and heat treatment
24Ac1-HT	2024 aluminum reinforced with 1 vol. % of activated nanocarbon and heat treatment
24Ac2-HT	2024 aluminum reinforced with 2 vol. % of activated nanocarbon and heat treatment
24Gn0.5-HT	2024 aluminum reinforced with 0.5 vol. % of graphene nanoplatelets and heat treatment
24Gn1-HT	2024 aluminum reinforced with 1 vol. % of graphene nanoplatelets and heat treatment

## Data Availability

The original contributions presented in the study are included within the article; further inquiries can be directed to the corresponding author.
